# Supplementary material for: Machine Learning Accelerates Crystallization for Structure Determination
Source: Angew Chem Int Ed Engl. 2026 May 4;65(25):e1218503. doi: 10.1002/anie.1218503 (PMC13266958; doi:10.1002/anie.1218503)

checkCIF/PLATON report

Structure factors have been supplied for datablock(s) lcz097

THIS REPORT IS FOR GUIDANCE ONLY. IF USED AS PART OF A REVIEW PROCEDURE FOR PUBLICATION, IT SHOULD NOT REPLACE THE EXPERTISE OF AN EXPERIENCED CRYSTALLOGRAPHIC REFEREE.

No syntax errors found. CIF dictionary Interpreting this report

Datablock: lcz097

```
Bond precision:   C-C = 0.0143 A          Wavelength=1.54184

Cell:            a=22.5622(2)     b=12.8850(1)       c=33.9620(3)
                 alpha=90         beta=103.233(1)    gamma=90
Temperature:     100 K

                Calculated                  Reported
Volume          9611.07(15)                 9611.06(15)
Space group     P 21/n                      P 1 21/n 1
Hall group      -P 2yn                      -P 2yn
Moiety formula  C15 H3 Ag3 F18 N6, C12 H10  C15 H3 Ag3 F18 N6, C12 H10
Sum formula     C27 H13 Ag3 F18 N6          C27 H13 Ag3 F18 N6
Mr              1087.04                     1087.04
Dx,g cm-3       2.254                       2.254
Z               12                          12
Mu (mm-1)       15.861                      15.861
F000            6240.0                      6240.0
F000'           6269.97
h,k,lmax        28,16,43                    28,15,41
Nref            20615                       19755
Tmin,Tmax       0.105,0.042                 0.412,1.000
Tmin'           0.017

Correction method= # Reported T Limits: Tmin=0.412 Tmax=1.000
AbsCorr = MULTI-SCAN
```

Data completeness= 0.958   Theta(max)= 78.280

R(reflections)= 0.0589(16454)   wR2(reflections)= 0.1611(19755)

S = 1.029   Npar= 1450

The following ALERTS were generated. Each ALERT has the format
test-name_ALERT_alert-type_alert-level.
Click on the hyperlinks for more details of the test.

Alert level C

```
PLAT213_ALERT_2_C Atom F025           has ADP max/min Ratio .....        3.2 prolat
PLAT213_ALERT_2_C Atom F03A           has ADP max/min Ratio .....        3.1 prolat
PLAT213_ALERT_2_C Atom F01V           has ADP max/min Ratio .....        3.3 prolat
PLAT213_ALERT_2_C Atom F03P           has ADP max/min Ratio .....        3.5 prolat
PLAT220_ALERT_2_C NonSolvent   Resd 1  F   Ueq(max)/Ueq(min) Range       3.3 Ratio
PLAT220_ALERT_2_C NonSolvent   Resd 2  F   Ueq(max)/Ueq(min) Range       3.1 Ratio
PLAT241_ALERT_2_C High   'MainMol' Ueq as Compared to Neighbors of       C04F Check
PLAT241_ALERT_2_C High   'MainMol' Ueq as Compared to Neighbors of       C04J Check
PLAT241_ALERT_2_C High   'MainMol' Ueq as Compared to Neighbors of       C04I Check
PLAT242_ALERT_2_C Low    'MainMol' Ueq as Compared to Neighbors of       C020 Check
PLAT242_ALERT_2_C Low    'MainMol' Ueq as Compared to Neighbors of       C03E Check
PLAT250_ALERT_2_C Large U3/U1 Ratio for <U(i,j)> Tensor(Resd    6)       2.4 Note
PLAT342_ALERT_3_C Low Bond Precision on  C-C Bonds ..............    0.01426 Ang.
PLAT360_ALERT_2_C Short  C(sp3)-C(sp3) Bond  C047     - C04J     .      1.42 Ang.
PLAT906_ALERT_3_C Large K Value in the Analysis of Variance ......     3.761 Check
PLAT911_ALERT_3_C Missing FCF Refl Between Thmin & STh/L=    0.600        77 Report
                1  1  0,   2  0  0,   2  1  0,  14 10  0,  -1  1  1,  14 13  1,
               16 12  1, -26  0  2,  -2  0  2,   1  8  2,   2  8  2,  10  0  2,
               15 12  2,   1  8  3,  -5 15  4,   0  8  4,   1  8  4,   2  0  4,
               -6 14  5,  -6 15  5,  -2  8  5,   1  8  5,  25  0  5,  -2  8  6,
               -1  8  6,   0  0  6,   0  8  6,   1  8  6,  -6  7  7, -11  6 10,
                   (   47 More Missing: see the .ckf listing file)
PLAT971_ALERT_2_C Check Calcd Resid. Dens.  1.10Ang From C04A           1.72 eA-3
PLAT971_ALERT_2_C Check Calcd Resid. Dens.  1.12Ang From F01V           1.58 eA-3
```

Alert level G

```
PLAT002_ALERT_2_G Number of Distance or Angle Restraints on AtSite         2 Note
PLAT003_ALERT_2_G Number of Uiso or U(i,j)  Restrained non-H-Atoms         7 Report
PLAT083_ALERT_2_G SHELXL Second Parameter in WGHT  Unusually Large     79.97 Why ?
PLAT142_ALERT_4_G s.u. on b - Axis Small or Missing .............    0.00010 Ang.
PLAT143_ALERT_4_G s.u. on c - Axis Small or Missing .............    0.00030 Ang.
PLAT172_ALERT_4_G The CIF-Embedded .res File Contains DFIX Records         1 Report
PLAT177_ALERT_4_G The CIF-Embedded .res File Contains DELU Records         1 Report
PLAT186_ALERT_4_G The CIF-Embedded .res File Contains ISOR Records         1 Report
PLAT192_ALERT_3_G A Non-default DELU Restraint Value for SecondPar    0.0200 Report
PLAT242_ALERT_2_G Low    'MainMol' Ueq as Compared to Neighbors of       C02N Check
PLAT242_ALERT_2_G Low    'MainMol' Ueq as Compared to Neighbors of       C03H Check
PLAT242_ALERT_2_G Low    'MainMol' Ueq as Compared to Neighbors of       C03K Check
PLAT242_ALERT_2_G Low    'MainMol' Ueq as Compared to Neighbors of       C03O Check
PLAT242_ALERT_2_G Low    'MainMol' Ueq as Compared to Neighbors of       C03T Check
PLAT242_ALERT_2_G Low    'MainMol' Ueq as Compared to Neighbors of       C03U Check
```

```
PLAT242_ALERT_2_G Low    'MainMol' Ueq as Compared to Neighbors of       C02T Check
PLAT242_ALERT_2_G Low    'MainMol' Ueq as Compared to Neighbors of       C030 Check
PLAT242_ALERT_2_G Low    'MainMol' Ueq as Compared to Neighbors of       C03D Check
PLAT242_ALERT_2_G Low    'MainMol' Ueq as Compared to Neighbors of       C03Q Check
PLAT242_ALERT_2_G Low    'MainMol' Ueq as Compared to Neighbors of       C03S Check
PLAT242_ALERT_2_G Low    'MainMol' Ueq as Compared to Neighbors of       C043 Check
PLAT242_ALERT_2_G Low    'MainMol' Ueq as Compared to Neighbors of       C03G Check
PLAT242_ALERT_2_G Low    'MainMol' Ueq as Compared to Neighbors of       C03I Check
PLAT242_ALERT_2_G Low    'MainMol' Ueq as Compared to Neighbors of       C03L Check
PLAT242_ALERT_2_G Low    'MainMol' Ueq as Compared to Neighbors of       C03N Check
PLAT242_ALERT_2_G Low    'MainMol' Ueq as Compared to Neighbors of       C03V Check
PLAT242_ALERT_2_G Low    'MainMol' Ueq as Compared to Neighbors of       C044 Check
PLAT335_ALERT_2_G Check Large C6 Ring C-C Range C020     -C036           0.19 Ang.
PLAT335_ALERT_2_G Check Large C6 Ring C-C Range C03E     -C04A           0.21 Ang.
PLAT434_ALERT_2_G Short Inter HL..HL Contact F00E     ..F01A     .       2.81 Ang.
                                             1-x,1-y,1-z  =      3_666 Check
PLAT434_ALERT_2_G Short Inter HL..HL Contact F012     ..F01C     .       2.83 Ang.
                                                x,-1+y,z  =      1_545 Check
PLAT434_ALERT_2_G Short Inter HL..HL Contact F018     ..F023     .       2.77 Ang.
                                                x,-1+y,z  =      1_545 Check
PLAT720_ALERT_4_G Number of Unusual/Non-Standard Labels ..........        201 Note
              Ag01    Ag02    Ag03    Ag04    Ag05    Ag06    Ag07    Ag08
              Ag09    F00A    F00B    F00C    F00D    F00E    F00F    F00G
              F00H    F00I    F00J    F00K    F00L    F00M    F00N    F00O
              F00P    F00Q    F00R    F00S    F00T    F00U    F00V    N00W
              N00X    N00Y    N00Z    F010    N011    F012    N013    N014
              F015    N016    N017    F018    N019    F01A    N01B    F01C
              N01D    F01E    N01F    N01G    F01H    N01I    N01J    F01K
              F01L    N01M    F01N    F01O    F01P    N01Q    F01R    F01S
              F01T    F01U    F01V    C01W    F01X    F01Y    F01Z    C020
              C021    C022    F023    F024    F025    C026    C027    C028
              C029    C02A    C02B    H02B    C02C    C02D    C02E    H02E
              C02F    C02G    H02G    F02H    C02I    H02I    C02J    C02K
              C02L    C02M    C02N    C02O    C02P    H02P    C02Q    C02R
              C02S    H02S    C02T    C02U    H02A    H02C    C02V    H02V
              C02W    H02W    F02X    F02Y    C02Z    C030    C031    H031
              C032    C033    F034    C035    H035    C036    C037    H037
              C038    F039    F03A    C03B    H03B    C03C    H03C    C03D
              C03F    C03G    C03H    C03I    C03J    H03A    H03D    C03K
              C03L    C03M    H03M    C03N    C03O    F03P    C03Q    C03R
              H03R    C03S    C03T    C03U    C03V    C03W    H03W    C03X
              H03X    C03Y    H03Y    C03Z    H03Z    F040    C041    H041
              C042    C043    C044    C045    H045    C046    H046    C047
              H04E    H04F    C04C    C03E    C04E    C04J    H04G    H04H
              C048    H04A    H04B    C049    H049    C04A    C04B    H04I
              C04D    H04J    C04F    H04C    H04D    C04G    H04K    C04I
              H04L
PLAT860_ALERT_3_G Number of Least-Squares Restraints ............          32 Note
PLAT883_ALERT_1_G Absent Datum for _atom_sites_solution_primary ..     Please Do !
PLAT910_ALERT_3_G Missing FCF Reflection(s) Below Theta(Min)[Deg]=       2.66 Note
               -1  0  1,
PLAT912_ALERT_4_G Missing # of FCF Reflections Above STh/L=  0.600        658 Note
PLAT941_ALERT_3_G Average HKL Measurement Multiplicity ..........         2.8 Low
PLAT952_ALERT_5_G Calculated (ThMax) and CIF-Reported Lmax Differ.          2 Units
PLAT958_ALERT_1_G Calculated (ThMax) and Actual (FCF) Lmax Differ.          2 Units
PLAT969_ALERT_5_G The 'Henn et al.' R-Factor-gap value ..........       3.008 Note
              Predicted wR2: Based on SigI**2  5.36 or SHELX Weight 15.66
```

```
PLAT978_ALERT_2_G Number C-C Bonds with Positive Residual Density.          0 Info
PLAT992_ALERT_5_G Repd & Actual _reflns_number_gt Values Differ by          2 Check
```

```
   0 ALERT level A = Most likely a serious problem - resolve or explain
   0 ALERT level B = A potentially serious problem, consider carefully
  18 ALERT level C = Check. Ensure it is not caused by an omission or oversight
  43 ALERT level G = General information/check it is not something unexpected

   2 ALERT type 1 CIF construction/syntax error, inconsistent or missing data
  42 ALERT type 2 Indicator that the structure model may be wrong or deficient
   7 ALERT type 3 Indicator that the structure quality may be low
   7 ALERT type 4 Improvement, methodology, query or suggestion
   3 ALERT type 5 Informative message, check
```

Validation response form

Please find below a validation response form (VRF) that can be filled in and pasted into your CIF.

```
# start Validation Reply Form
_vrf_PLAT213_lcz097
;
PROBLEM: Atom F025            has ADP max/min Ratio .....        3.2 prolat
RESPONSE: ...
;
_vrf_PLAT220_lcz097
;
PROBLEM: NonSolvent   Resd 1  F   Ueq(max)/Ueq(min) Range        3.3 Ratio
RESPONSE: ...
;
_vrf_PLAT241_lcz097
;
PROBLEM: High   'MainMol' Ueq as Compared to Neighbors of       C04F Check
RESPONSE: ...
;
_vrf_PLAT242_lcz097
;
PROBLEM: Low    'MainMol' Ueq as Compared to Neighbors of       C020 Check
RESPONSE: ...
;
_vrf_PLAT250_lcz097
;
PROBLEM: Large U3/U1 Ratio for <U(i,j)> Tensor(Resd    6)        2.4 Note
RESPONSE: ...
;
_vrf_PLAT342_lcz097
;
PROBLEM: Low Bond Precision on  C-C Bonds ..............    0.01426 Ang.
RESPONSE: ...
;
_vrf_PLAT360_lcz097
;
PROBLEM: Short  C(sp3)-C(sp3) Bond  C047     - C04J     .       1.42 Ang.
RESPONSE: ...
;
_vrf_PLAT906_lcz097
```

```
;
PROBLEM: Large K Value in the Analysis of Variance ......      3.761 Check
RESPONSE: ...
;
_vrf_PLAT911_lcz097
;
PROBLEM: Missing FCF Refl Between Thmin & STh/L=    0.600         77 Report
RESPONSE: ...
;
_vrf_PLAT971_lcz097
;
PROBLEM: Check Calcd Resid. Dens.  1.10Ang From C04A            1.72 eA-3
RESPONSE: ...
;
# end Validation Reply Form
```

It is advisable to attempt to resolve as many as possible of the alerts in all categories. Often the minor alerts point to easily fixed oversights, errors and omissions in your CIF or refinement strategy, so attention to these fine details can be worthwhile. It is up to the individual to critically assess their own results and, if necessary, seek expert advice.

PLATON version of 17/09/2025; check.def file version of 17/09/2025

---

duplicate check

No duplication found

Datablock lcz097 - ellipsoid plot

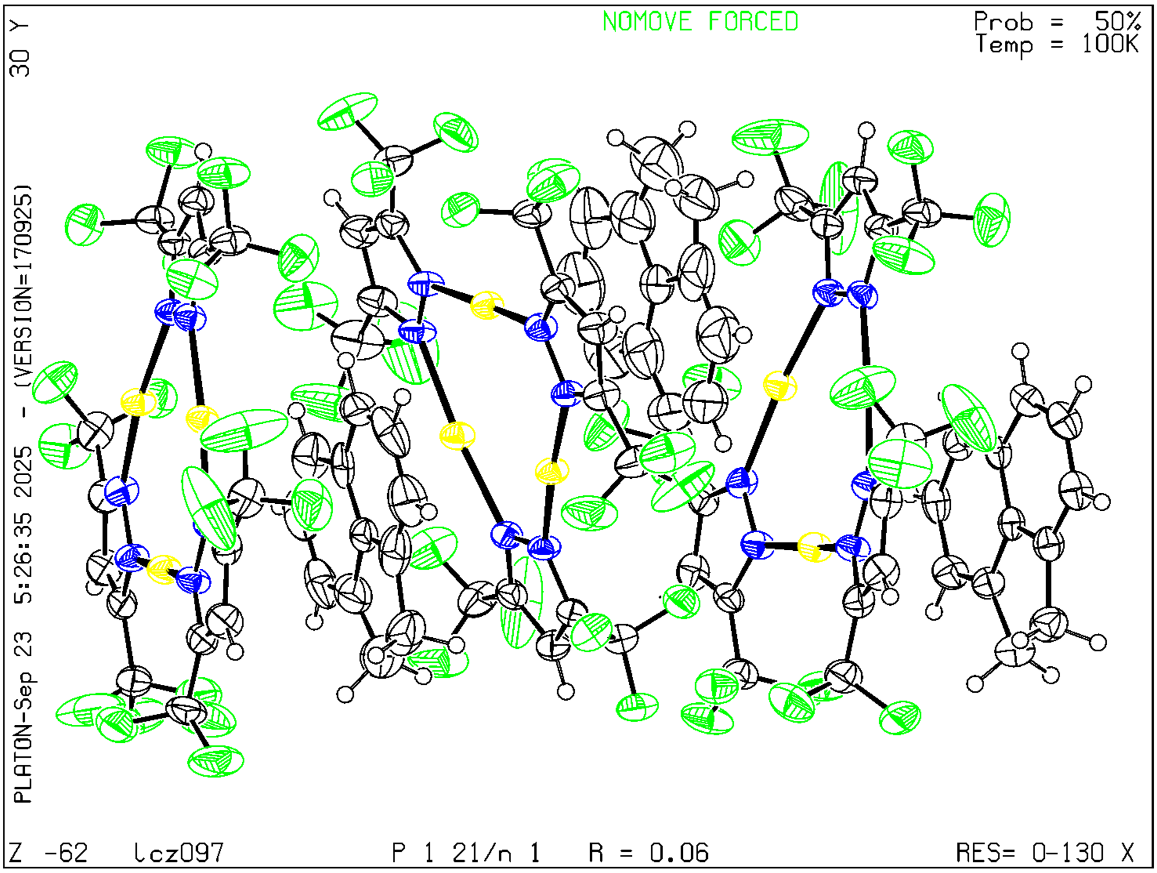

Supplement: Supplementary file 2 — Supporting File 2: anie72462‐sup‐0002‐DataFile.zip. [file ANIE-65-e1218503-s002.zip › supplementary_materials_crystallographic_data submit.zip!/cif_and_checkcif(1-114)/Ag3Pz3_97/Ag3Pz3_97_checkcif.pdf]
